# Sentinels in the shadows: Exploring *Toxoplasma gondii* and other Sarcocystidae parasites in synanthropic rodents and their public health implications

**DOI:** 10.1016/j.ijppaw.2024.100939

**Published:** 2024-04-17

**Authors:** Filippo Maria Dini, Monica Caffara, Alice Magri, Alessia Cantori, Valentina Luci, Antonio Monno, Roberta Galuppi

**Affiliations:** Department of Veterinary Medical Sciences (DIMEVET), Alma Mater Studiorum University of Bologna, Via Tolara di Sopra 50, 40064, Ozzano Emilia, BO, Italy

**Keywords:** Toxoplasmosis, Zoonosis, Rodentia, Apicomplexa, *Hammondia hammondi*, *Besnoitia* sp, *Sarcocystis gigantea*

## Abstract

Synanthropic rodents play a crucial role in maintaining the life cycle of *Toxoplasma gondii* in anthropized regions and can serve as indicators of environmental oocyst contamination. This investigation aimed to explore the occurrence of *T. gondii* infection within synanthropic rodent populations using a molecular diagnostic technique targeting the 18S rDNA gene, which is generic for Coccidia, with subsequent specific PCR confirmation. We examined 97 brown rats (*Rattus norvegicus*), 67 black rats (*R. rattus*), 47 house mice (*Mus musculus*), and 1 common shrew (*Sorex araneus*). PCR tests were conducted on the brain, heart, and tongue tissues. PCR tested positive in at least one of the examined tissues in 26 *R. norvegicus* (26.8%), 13 *R. rattus* (19.4%), and 13 *M. musculus* (27.6%). Sequencing comparisons by BLAST allowed us to identify four different species of cyst-forming Apicomplexa. In particular, *T. gondii* DNA was detected in 13 (6.1%) rodents, *Hammondia hammondi* (including *H. hammondi*-like organisms) in 36 (17%) subjects, *Besnoitia* sp. (in two cases identified as *B. besnoiti*) in 8 (3.7%), and *Sarcocystis gigantea* in two (0.94%). Rodents from peri-urban and urban environments can act as indicators of environmental contamination by oocysts of apicomplexan parasites with cats as definitive hosts, such as *T. gondii*, *H. hammondi*, and *S. gigantea*, the latter of which has never been previously recorded in rodents. Moreover, the presence of *B. besnoiti*, a parasite with an unidentified definitive host in Europe, sheds light on the potential role of these hosts as infection sentinels.

## Highlights

• Molecular prevalence of Sarcocystidae in synanthropic rodents is assessed.
• *Hammondia hammondi* and *Toxoplasma gondii* are the most prevalent Sarcocystidae.
• *Besnoitia besnoiti* first detection in rodents in Italy.
• *Sarcocystis gigantea* may infect also rodents in anthropized settings.

## Graphical abstract

**Figure:**
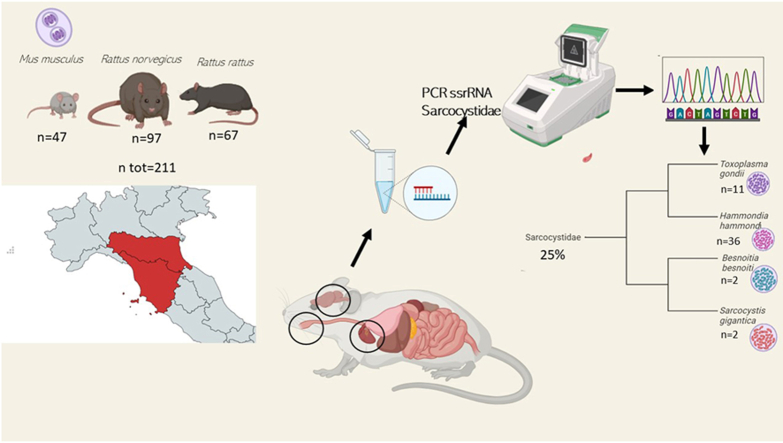

## 1 Introduction

Synanthropism, as defined by Klegarth (2016), encompasses the behaviour of wildlife (or flora) thriving within the shared ecosystems of humans. This behaviour, in turn, drives an increase in population density, reproduction rates, and survival advantages among these synanthropic species. Conversely, their territorial range diminishes due to their reliance on centralized anthropogenic resources (Gehrt et al., 2011; Hulme-Beaman et al., 2016).

From the time of the Neolithic Revolution, human activities have led to profound and enduring impacts on the natural environment. This process of settling down and adopting agricultural practices created a stable ecological niche that ensured sustenance over extended periods. Consequently, this environment began to attract initial rodent populations, as documented by Frynta et al. (2005) and Cucchi et al. (2005), which in turn drew the interest of subsequent cat populations (Krajcarz et al., 2022). Through the passing decades and centuries, these modest human settlements gradually expanded into villages and towns, forming the earliest instances of urban settings inhabited by synanthropic species (Baumann, 2023).

In this context, the establishment of a predator-prey relationship has given rise to the development of a peri-domestic life cycle of predation-associated parasites (Mendoza Roldan and Otranto, 2023). The process of predation stands out as one of the most effective mechanisms for facilitating the transmission of parasites, offering a direct pathway for the parasite to fulfil its life cycle within the trophic chain (Johnson et al., 2010; Médoc and Beisel, 2011). Parasites transmitted through trophic interactions have, in various instances, undergone adaptations to optimize predation through manipulation of their host preys (Seppälä et al., 2008). A pertinent illustration of this phenomenon can be observed in *Toxoplasma gondii* (Eucoccidiorida: Sarcocystidae), which relies on the predatory behaviour of felines (definitive hosts of the parasite), that include the consumption of small rodents and other prey species, to successfully conclude its life cycle (Vyas et al., 2007; Dubey et al., 2021). The reproductive fitness of *Toxoplasma* is intricately linked to the predation patterns exhibited by felids. Disruption of the innate aversion mechanism, caused by the interaction of the parasite with the SNC of the host, heighten predation rates, thereby increasing the reproductive fitness of the parasite. This stance aligns with the ‘behavioural manipulation’ hypothesis, postulating that *T. gondii* can induce alterations in host behaviour that directly contribute to the enhancement of their own reproductive success, as proposed by Vyas et al. (2007) and Webster (2007).

Rodents play for this reason a crucial part in upholding the lifecycle of *T. gondii* and influencing the spread of toxoplasmosis. This significance is particularly pronounced in species residing near human settlements. The establishment of an infection transmission cycle via rodents (and other small preys) results in the release of millions of unsporulated oocysts by cats, that can therefore be found in various environmental matrices where, upon sporulation, become infectious and can remain viable up to several years (Dubey et al., 2021). Consequently, this process heightens the risk of infection for all hosts of the parasite in the ecosystem, most notably humans within these habitats (Mercier et al., 2013).

Apart from their crucial role in maintaining the life cycle of *T. gondii* in anthropized regions, synanthropic rodents, due to their feeding behaviour that predominantly facilitate oral transmission of sporulated oocysts within the environment, can be regarded as indicative of environmental oocyst contamination. Consequently, the finding of *T. gondii* in wild rodent populations might reflect the dissemination of the parasitic environmental phase within a specific geographical area (Dubey et al., 2021).

The main aim of this study is to investigate the prevalence of *T. gondii* infection among synanthropic rodent populations in a specific area of Italy that has been previously studied to assess prevalence rates in animals and humans. These earlier studies revealed a consistent presence of the parasite in the region (Dini et al., 2023a, Dini et al., 2023b; Dini et al., 2024a, Dini et al., 2024b). The central aim of this study is to ascertain the potential environmental significance of the parasite within urbanized settings. This is achieved by evaluating the occurrence of infections in rodents residing in the peri-domestic environment, utilizing molecular diagnostic techniques.

## 2 Materials and methods

**Fig. 1:**
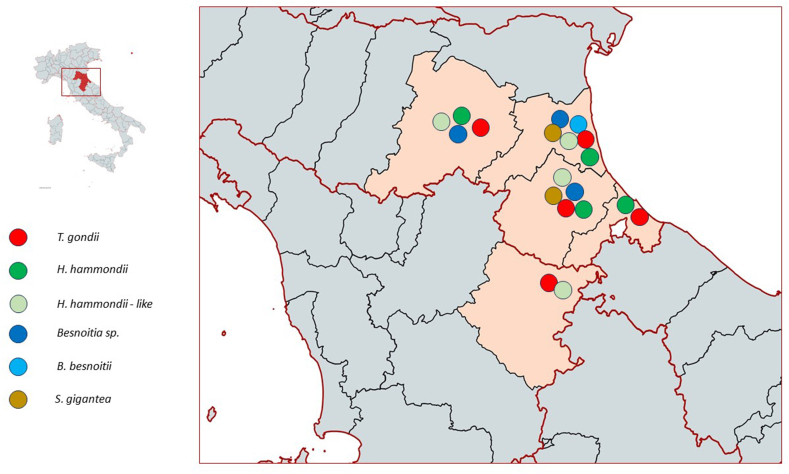
Geographical distribution of PCR positive rodents.

From June 2019 to March 2023, 212 carcasses of adults or subadults peridomestic rodent were collected and stored at −20 °C by professional rodent control services during pest control programs from urban and rural areas in the provinces of Ferrara, Forlì-Cesena, Ravenna, Bologna (Emilia Romagna Region) and Arezzo (Toscana Region) (Fig. 1). In detail, 97 brown rats (*Rattus norvegicus*), 67 black rats (*Rattus rattus*), 47 house mouse (*Mus musculus*) and 1 common shrew *(Sorex araneus*) were sampled. The carcasses were identified morphologically according to CDC (2006); sex and weight of each rodents were recorded. Sampling was performed with sterile surgical instruments and, according to the state of the carcasses, 25–50 mg of tissue were collected from heart, 25–200 mg from brain and 25 mg from tongue muscle. Samples were placed in sterile 1.5 ml tubes and stored at −20 °C until DNA extraction. In total, 209 brains (due to poor condition of 3 *R. rattus*), 212 tongue, and 180 heart samples (poor condition of 19 *R norvegicus*, 10 *R. rattus*, and 3 *M. musculus*) were collected.

Genomic DNA was purified using Pure Link ® Genomic DNA Mini kit (Invitrogen by Thermo Fisher), according to the manufacturer's protocol. Initial end-point PCR targeting 18S rDNA gene of Coccidia was performed on all the samples with the primers COC-1 and COC-2 as described by Ho et al. (1996) following some modification by Hornok et al. (2015). Briefly, a reaction volume of 25 μl, containing 12.5 μl 2x Dream Taq Hot Start Green PCR Master Mix (Thermo Scientific), 9.5 μl ddH2O, 0.25 μl (1 μM final concentration) of each primer, and 2.5 μl template DNA were used. For amplification, an initial denaturation step at 94 °C for 10 min was followed by 40 cycles of denaturation at 94 °C for 30 s, annealing at 54 °C for 30 s and extension at 72 °C for 30 s. Final extension was performed at 72 °C for 10 min. In all the PCRs, sterile water was included as negative control.

An additional species-specific PCR have been carried out to validate the species identification obtained through sequencing the 18S rDNA fragment amplified with COC primers. Specifically, for *Besnoitia* sp. specimens, we employed an end-point PCR targeting 231 bp of the ITS-1 rDNA following the method of Cortes et al. (2007).

To confirm the *Sarcocystis gigantea* samples, an end-point PCR assay was used with *Sarcocystis* genus-specific primers: sarF (TGGCTAATACATGCGCAAATA) (Vangeel et al., 2007) and sarcoREV (AACCCTAATTCCCCGTTA) (Chiesa et al., 2013), amplifying an expected region of 240 bp following the protocol reported by Rubiola et al. (2020).

Finally, to confirm *Toxoplasma gondii* and *Hammondia hammondi* positive samples, we employed a semi-nested PCR targeting the ITS rDNA as described by Sreekumar et al. (2005).

Amplifications were performed in a T-personal thermal cycler (Biometra, Göttingen, Germany). The PCR products were electrophoresed on 1.5% agarose gel stained with SYBR Safe DNA Gel Stain (Thermo Fisher Scientific, Carlsbad, CA, USA) in 0.5 × TBE. For sequencing, the amplicons were excised and purified by Nucleo-Spin Gel and PCR Clean-up (Mackerey-Nagel, Düren, Germany), and sequenced with an ABI 3730 DNA analyzer (StarSEQ, Mainz, Germany).

The trace files were assembled with Contig Express (VectorNTI Advance 11 software, Invitrogen, Carlsbad, CA, USA), and the consensus sequences were compared with published data by BLAST tools (https://blast.ncbi.nlm.nih.gov/Blast.cgi). Sequence alignments were carried out by BioEdit 7.2.5 (Hall, 1999), while p-distance and maximum-likelihood (ML) tree (GTR + G + I substitution model for both genes and bootstrap of 1000 replicate) were calculated by MEGA 7 (Kumar et al., 2016).

Student t-test was used to compare the average weight of male and female; Pearson's χ2 test was used to associate sex with prevalence data. Differences were considered significant when P ≤ 0.05. The Sample Size Calculator (https://www.surveysystem.com/sscalc.htm) was used to calculate 95% confidence intervals (CIs) for the observed prevalence values.

## 3 Results

Among the 212 rodents collected, 133 were males while 78 were females as detailed in Table 1 together with the species sex and weight. In each species no significant weight differences between sexes were detected by Student's t-test. The single shrew collected, was a male weighing 4 g.

**Table 1:** Descriptive statistics and PCR results.

Species	n.	Sex	n. (%)	Weight (g)			PCR positive (%)	CI 95%
				min	max	median		
*Rattus norvegicus*	97	M	64 (66%)	28.5	490	228.5	12 (18.7%)	[9.19–28.31]
		F	33 (34%)	45	440	188	14 (42.4%)	[25.56–59.28]

*Rattus rattus*	67	M	41 (61.2%)	6	165	90	8 (19.5%)	[7.38–31.64]
		F	26 (38.81%)	5.2	195	106	5 (19.2%)	[4.08–34.4]

*Mus musculus*	47	M	28 (59.6%)	4.6	70	14.6	9 (32.1%)	[14.84–49.44]
		F	19 (40.4%)	4.9	25	16	4 (21%)	[2.72–39.38]

Overall, 53 rodents (25%) were PCR positive in at least one of the examined tissues; splitting the results: *R. norvegicus* 26 out of 97 (26.8%, CI 95% = 7.99–35.61), *R. rattus* 13 out of 67 (19.4%, CI 95% = 9.93–28.87) while *M. musculus* 13 out of 47 (27.6%, CI 95% = 14.87–40.45). In Table 1 the PCR results are also reported in relation to sex. Notably, in *R. norvegicus*, females showed a significantly higher positivity rate (42.4%) than males (18.7%) (Yates-corrected chi-square: 5.7; p = 0.0243), while no significant sex differences were observed for *R. rattus* and *M. musculus.*

Sequences comparison by BLAST allows to identify four different species of cyst-forming Apicomplexa (Sarcocystidae): *Toxoplasma gondii, Hammondia hammondi* (including *H. hammondi*-like organism), *Besnoitia* sp. and *Sarcocystis gigantea*.

In particular *T. gondii* was detected in 13 (6.1%) rodents, (similarities 99.7%–100%), *H. hammondi* (and *H. hammondi*-like organism) in 36 (17%) subject (similarities 99.3%–100%), *Besnoitia* sp. was found in 8 rodents (3.7%), with low sequence similarity (94%–95%). Notably, only two samples showed 100% similarity with *B. besnoiti* from cattle (XR 003828656-59, KJ746531, JF314861). Finally, *S. gigantea* was detected in two heart samples of *R. norvegicus*, constituting 0.94% of the total rodent population and 2% of the *R. norvegicus* samples. These samples displayed 100% sequence similarity with sequences of *S. gigantea* available in GenBank (MT026574, MK420020, OP550293, KC209733).

In the context of host species differentiation, within *R. norvegicus*, the most frequently encountered cyst-forming coccidia was *H. hammondi* (and *H. hammondi*-like) with a prevalence of 17.5%, followed by *Besnoitia* sp. at 7.2%. Among the latter only one sequence showed 100% similarity with *B. besnoiti*. Trailing behind in terms of prevalence were *T. gondii* at 4% and *Sarcocystis gigantea* at 2%. In *R. rattus*, only two Apicomplexan species were molecularly identified in the analysed tissues: *H. hammondi*/*H. hammondi-like* (11.9%), and *T. gondii* (9%). Concerning *M. musculus*, the most prevalent was *H. hammondi/H. hammondi*-like (23.4%), followed by *T. gondii* (4.2%). Intriguingly, in one instance, *B. besnoiti* was detected in a heart sample with 100% sequence similarity.

Regarding the distribution of positive matrices in relation to parasites, *H. hammondi/H. hammondi*-like DNA was more frequently observed in CNS and heart samples; *T. gondii* was equally distributed among all matrices, while *B. besnoiti* occurred only in heart samples.

Co-infections (referred to the detection of DNA of more than one parasite in the same host) were only observed in *Rattus* spp. and *M. musculus*. Among *Rattus* species, one *R. norvegicus* and one *R. rattus* exhibited co-infection by *T. gondii/H. hammondi + H. hammondi*-like. Additionally, two *R. norvegicus* showed co-infection by *H. hammondi*-like and *Besnoitia* sp., while one *R. norvegicus* exhibited co-infection by *H. hammondi-*like and *Sarcocystis gigantea*. In the case of *Mus musculus*, co-infection by *T. gondii* and *H. hammondi*-like was detected. The sole analysed *S. araneus* was found positive for *T. gondii* (100% sequence similarity with KX007999-KX008033) in heart tissue.

The results of the Sarcocystidae generic PCR, conducted on the 18S rDNA, are presented in Table 2, Table 3, Table 4, along with species confirmation achieved through specific PCR assays (ITS rDNA for *T. gondii and H. hammondi* and *B. besnoiti* and 18S rDNA for *S. gigantea*).

**Table 2:** List of the 26 *R. norvegicus* positive at 18S rDNA PCR and sequencing along with species confirmation achieved through specific PCR assays.

ID number	Sex	Weight	Brain	Tongue	Heart
20	F	390	–	–	*Besnoitia* sp.
82	M	40	*H. hammondi*-like	–	–
83	F	320	–	*H. hammondi*-like	–
84	M	155	–	–	*Besnoitia* sp.
87	F	320	*T. gondii*^a^	–	–
135	M	74,5	–	*Besnoitia* sp.	na
136	F	240	*H. hammondi*^a^	–	na
137	F	370	–	*Besnoitia* sp.	–
140	M	265	*H. hammondi*^a^	–	–
144	M	470	–	*Besnoitia besnoiti*^b^	–
216	M	191	*H. hammondi*^a^	*H. hammondi*-like	*H. hammondi*^a^
217	F	156	–	–	*S. gigantea*^c^
219	F	185,7	*H. hammondi*^a^	–	–
220	F	86,19	*H. hammondi*^a^	–	*H. hammondi*-like
221	M	36,6	–	*H. hammondi*-like	*H. hammondi*-like
3	F	180	–	*H. hammondi*-like	*H. hammondi*^a^
4	F	314	*H. hammondi*^a^	*H. hammondi*-like	*H. hammondi*^a^
19	M	96	*T. gondii*^a^	–	–
22	F	45	*H. hammondi*^a^	–	*T. gondii*^a^
25	F	80	*H. hammondi*^a^	–	*Besnoitia* sp.
26	M	294,5	*H. hammondi*^a^	–	*S. gigantea*^c^
69	M	48	*H. hammondi*^a^	–	–
71	F	220	*Besnoitia* sp.	–	*H. hammondi*^a^
78	F	94,7	*H. hammondi*^a^	–	*H. hammondi*^a^
79	M	82	–	–	*H. hammondi*^a^
81	M	132	–	–	*T. gondii*^a^

a species confirmed by ITS specific PCR.

b species confirmed by ITS specific PCR.

c species confirmed by 18S specific PCR.

**Table 3:** List of the 13 *R. rattus* positive at 18S rDNA PCR and sequencing along with species confirmation achieved through specific PCR assays.

ID number	Sex	weight	Brain	Tongue	Heart
76	F	5,2	–	*T. gondii*^a^	–
77	F	6	–	–	*H. hammondi*^a^
148	M	91	–	*T. gondii*^a^	–
215	F	82	*H. hammondi*^a^	*T. gondii*^a^	*H. hammondi*^a^
218	F	97	*H. hammondi*^a^	–	–
1	M	104	*H. hammondi*^a^	*H. hammondi*^a^	*H. hammondi*^a^
2	M	90	*H. hammondi*-like	*H. hammondi*-like	*H. hammondi*-like
21	F	139,22	*T. gondii*^a^	–	–
30	M	162,95	–	*T. gondii*^a^	–
31	M	141,3	*H. hammondi*^a^	–	*H. hammondi*-like
38	M	59,6	–	–	*H. hammondi*^a^
51	M	140	*H. hammondi*^a^	*H. hammondi*-like	*H. hammondi*^a^
75	M	45	–	–	*T. gondii*^a^

a species confirmed by ITS specific PCR.

**Table 4:** List of the 13 *M. musculus* positive at 18S rDNA PCR and sequencing along with species confirmation achieved through specific PCR assays.

ID number	Sex	Weight	Brain	Tongue	Heart
101	F	17	–	–	*T. gondii*^a^
109	F	25	–	*H. hammondi*-like	–
111	M	17	–	–	*Besnoitia besnoiti*^b^
112	F	22	–	–	*H. hammondi*^a^
113	F	15	*H. hammondi*-like	–	–
115	M	14	*H. hammondi*-like	–	*H. hammondi*^a^
117	M	23	*H. hammondi*-like	–	–
119	M	7,9	–	*H. hammondi*-like	*H. hammondi*-like
120	M	7	*H. hammondi*^a^	–	*H. hammondi*-like
122	M	14,7	*H. hammondi*-like	–	–
7	M	21	–	–	*H. hammondi*-like
32	M	9,7	–	*H. hammondi*-like	na
80	M	18,5	*T. gondii*^a^	*H. hammondi*-like	–

a species confirmed by ITS specific PCR.

b species confirmed by ITS specific PCR.

The alignment of all *T. gondii* obtained in the present study (plus the *T. gondii* type I, RH reference strain) with *H. hammondi/H. hammondi*-like showed the presence of two transitions C/T and A/G as the only differences between the two species over 297 bp of the 18S rDNA. Moreover, the A/G transition further separate the “true” *H. hammondi* from the *H. hammondi*-like group. The p-distance observed between *T. gondii* and the group of *H. hammondi* is very low ranging from 0% to 0.1%, due to the low genetic resolution of the 18S rDNA.

The ML tree obtained (tree not reported), despite with a low bootstrap support, showed 3 separated clusters, one composed by our *T. gondii* together with the reference strain, the second by the “true” *H. hammondi* (having 100% identity with AH008381) and the latter by *H. hammondi*-like group.

The sequences obtained in this study have been deposited in GenBank under the accession numbers PP500534-PP500612 (18S rDNA) and PP502867-PP502868 (ITS rDNA).

## 4 Discussion

*Rattus* spp. and *M. musculus* exemplify species capable of coexisting within anthropogenically influenced environments. This coexistence raises concerns for potential human health risks owing to the close proximity between these species and human habitats. Rodents constitute a notably significant group of mammals, particularly in terms of serving as reservoirs for various pathogens, some of which are of zoonotic concern (Han et al., 2015). Their biological attributes, characterized by elevated reproductive rates, opportunistic behaviours, adaptability, and worldwide distribution, position them strategically, thereby enhancing the likelihood of disease transmission among wildlife, domestic animals, and human populations (Han et al., 2015; Luis et al., 2013)

Within the scope of this research, we examined the occurrence of Sarcocystidae infections in synanthropic rodents using a broad-spectrum PCR assay targeting the 18S rRNA of Coccidia, and subsequent specific PCR confirmation. This molecular method, involving a single PCR assay followed by Sanger sequencing, enabled us to reveal the presence of various protozoan species, namely *T. gondii*, *H. hammondi/H. hammondi*-like, *Besnoitia* sp., and *S. gigantea*, within the studied host species (*R. norvegicus*, *R. rattus*, *M. musculus*, and *S. araneus*).

In the current study, *T. gondii* DNA was detected in all the rodent host species, with an overall prevalence rate of 6.1%. Notably, among these species, *R. rattus* exhibited the highest infection rate, with 9% prevalence. The worldwide seroprevalence is approximately 6%, with the highest rates recorded in Africa (24%) and South America (18%), and the lowest in Europe (1%) (Galeh et al., 2020). It's important to note that serological tests cannot definitively predict the presence/absence of the parasite. This limitation has been previously observed (Dubey, 2022), as viable *T. gondii* has been isolated from rodents that tested seronegative (AraùJo et al., 2010). Hence, molecular studies appear to offer more epidemiological reliability.

Rodents play a pivotal role in the perpetuation of the *T. gondii* life cycle and the epidemiology of toxoplasmosis. They are recognized as reservoirs and carriers of the disease, serving as the primary source of infection for cats and their related species (Dabritz et al., 2008). This role becomes particularly significant in species inhabiting close proximity to human habitats due to the profound implications for both the environment and human health. The establishment of the infection transmission cycle through rodents results in the release of oocysts from infected felids, leading their dissemination into environment. Consequently, this amplifies the infection risk for various hosts including humans (Mercier et al., 2013). The importance of rodents in maintaining the lifecycle of *T. gondii* has been further strengthened and highlighted following studies involving the neuroanatomical interaction of chronical established CNS cysts in the behavioural pattern of these hosts. Numerous studies indicate that *T. gondii* alters rodent behaviour, making them more susceptible to predation by cats (Webster, 2007). These alterations include increased activity, reduced neophobia (fear of novelty), and decreased predator vigilance (Webster, 1994, 2001, 2007; Webster and Brunton, 1994; Berdoy et al., 1995; Lamberton et al., 2008). These changes likely facilitate parasite transmission from the intermediate host to the feline host. (Berdoy et al., 2000; Vyas et al., 2007; Kaushik et al., 2014).

*Hammondia hammondi* (including *H. hammondi*-like), molecularly found in the 17% of the analysed rodents, is a non-zoonotic coccidian parasite that bears a close resemblance to *T. gondii* (Frenkel and Dubey, 1975). The life cycles of these two parasites also share similarities like in the definitive hosts (Felidae) but differently from *T. gondii*, *H. hammondi* follows an obligatory two-host lifecycle, consequently, only sporulated oocysts are infective to rodents, and solely bradyzoite cysts are infective to cats (Frenkel and Dubey, 2000). Intermediate hosts include mice (*Mus musculus*), rats (*Rattus norvegicus*), and other rodents but also, rabbits, hamsters, goats, dogs, and pigs (Shimura and Ito, 1987). The prevalence of *H. hammondi* remains undisclosed in both definitive and intermediate hosts. Only few research reported the sporadically presence of *H. hammondi* in cat and dog faeces (Schares et al., 2005, 2008; Dubey et al., 2013) and in intermediate host tissues (Cabral et al., 2021).

In our study the consistent presence of *H. hammondi/H. hammondi*-like DNA in the rodent hosts are not comparable with other data, since no previous report are, to the best of our knowledge, present in available literature.

The distinction between *T. gondii* and *H. hammondi* is of paramount importance, particularly because the latter lacks zoonotic relevance, despite its close genetic relationship. In our investigations, the 18S rDNA despite being highly conserved, emerged as a sensitive genetic marker within the Apicomplexa subphylum, able to distinguish between the two parasites. Notably, there are only a few nucleotide variations between the complete 18S rDNA sequences of *T. gondii* and *H. hammondi* (Jenkins et al., 1999). Within our sequences, a single nucleotide variation, represented by C/T and A/G transition, effectively separated *T. gondii* from *H. hammondi* samples, leading to the formation of two distinct clusters among our positive sample set. Moreover, the latter transition (A/G) further delineated these samples into two sub-clusters, “*H. hammondi*” and “*H. hammondi-*like organism”. In light of the limited knowledge regarding the global population structure of *H. hammondi*, it remains conceivable that yet undiscovered lineages of *H*. *hammondi* may exist, displaying potential genetic diversity (Schares et al., 2021).

Within our study, 3.7% of the rodents exhibited DNA sequences associated with *Besnoitia* sp. However, it's noteworthy that only two sequences matched with 100% identity to *B. besnoiti*. The other specimens exhibited a sequence similarity of 94–95% with sequences of *Besnoitia* spp. available in GenBank, and lower similarity with other Sarcocystidae parasites. This outcome renders the accurate assignment of a species identification for these positive samples challenging, thereby impeding our ability to make informed epidemiological inferences.

This parasite is responsible for bovine Besnoitiosis, a chronic and debilitating disease that has been causing significant economic losses in cattle and has been considered endemic in Italy since 2011 (Gentile et al., 2012). In Europe, no definitive hosts for this parasite have been identified (Basso et al., 2011). The presence of parasite cysts in the skin suggests that the primary way of transmission is likely mechanical, with hematophagous flies serving as the carriers of the parasite (Bigalke, 1968). The intriguing aspect is the molecular identification of this parasite in the internal organs of rodent hosts, particularly in the heart, which raises questions about the epidemiology and potential host range of this Sarcocystidae. Notably the two subjects in which *B. besnoiti* was detected, were collected from two areas where a consistent population of domestic ruminant is present. This discovery hints the possibility that rodent hosts could serve as competent hosts in the life cycle of *Besnoitia*. It's worth noting that other *Besnoitia* species, such as *B. jellisoni* and *B. wallacei*, have been previously described in rodents but have not been documented in Europe until now (Alvarez-García et al., 2013).

Finally, our research has unveiled the occurrence of *S. gigantea* in the heart muscles of two *R. norvegicus*. The genus *Sarcocystis* comprises apicomplexan protozoa forming cysts, with a life cycle obligatorily entailing two hosts (Dubey et al., 2015). These cysts are typically located in the striated muscles of herbivorous or omnivorous intermediate hosts, while carnivores serve as the definitive hosts. Remarkably, more than 40 *Sarcocystis* species have been identified to use rodents as their intermediate hosts, including *S. microti* (Votýpka et al., 1998; Mugridge et al., 1999), *S. muris* (Mugridge et al., 1999; Gajadhar et al., 1991), *S. myodes* (Rudaitytė-Lukošienė et al., 2022), and *S. ratti* (Zeng et al., 2020; Prakas et al., 2019). An apparent gap in research exists concerning the global prevalence of *Sarcocystis* spp. in small mammals. Researchers have proposed that infection rates of various *Sarcocystis* species are contingent upon factors such as the specific parasite species, intermediate host species, geographical region, as well as the presence and abundance of definitive hosts within the studied area (Rudaitytė-Lukošienė et al., 2022; Hu et al., 2022). *Sarcocystis gigantea* infection is considered to be mildly pathogenic yet relatively common in sheep, with the cat as the definitive host (Dubey et al., 2015). While *S. gigantea* has not been documented in rodent hosts to date, recent findings have indicated its capacity to cause infections in horses, which serve as non-specific intermediate hosts (Veronesi et al., 2020). This observation suggests that in peri-urban settings where definitive hosts, such as cats, are abundant and the parasite is disseminated within the environment, non-specific intermediate hosts, such as rodents, may potentially develop bradyzoite cysts within their muscle tissue. Our findings support the hypothesis that certain *Sarcocystis* spp. may possess a broader range of intermediate hosts than was previously recognized (Veronesi et al., 2020).

## 5 Conclusions

In conclusion, the findings of this study highlight that synanthropic rodents sampled in urban and peri-urban environments serve as valuable indicators of environmental contamination by oocysts of apicomplexan parasites with cat as definitive host. This applies not only to parasites like *T. gondii* and *H. hammondi*, which are closely related apicomplexans with distinct epidemiological implications but both having cat-rodent cycle as a robust framework. This pattern is also applicable for *S. gigantea*, recovered in the hearts of two *R. norvegicus*, and recognizing cats as the definitive host. This species has never been recorded previously in rodents. Lastly, the presence of *B. besnoiti* in *R. norvegicus* and *M. musculus*, parasite with an unidentified definitive host in Europe, sheds light on the potential role of these hosts as infection sentinels.

## CRediT authorship contribution statement

**Filippo Maria Dini:** Writing – original draft, Methodology, Investigation. **Monica Caffara:** Writing – review & editing, Methodology. **Alice Magri:** Investigation, Methodology. **Alessia Cantori:** Methodology, Investigation. **Valentina Luci:** Methodology, Investigation. **Antonio Monno:** Investigation. **Roberta Galuppi:** Writing – review & editing.

## Declaration of competing interest

The authors declare that they have no known competing financial interests or personal relationships that could have appeared to influence the work reported in this paper.
